# Machine Learning-Based Pressure Ulcer Prediction in Modular Critical Care Data

**DOI:** 10.3390/diagnostics12040850

**Published:** 2022-03-30

**Authors:** Petr Šín, Alica Hokynková, Nováková Marie, Pokorná Andrea, Rostislav Krč, Jan Podroužek

**Affiliations:** 1 Department of Burns and Plastic Surgery, Faculty Hospital Brno and Faculty of Medicine, Masaryk University, Jihlavská 20, 625 00 Brno, Czech Republic; p.sin@seznam.cz; 2 Department of Physiology, Faculty of Medicine, Masaryk University, Kamenice 5, 625 00 Brno, Czech Republic; majka@med.muni.cz; 3 Department of Health Sciences, Faculty of Medicine, Masaryk University, Kamenice 5, 625 00 Brno, Czech Republic; apokorna@med.muni.cz; 4 Institute of Computer Aided Engineering and Computer Science, Faculty of Civil Engineering, Brno University of Technology, Veveří 331/95, 602 00 Brno, Czech Republic; rostislav.krc@gmail.com (R.K.); podrouzek.j@fce.vutbr.cz (J.P.)

**Keywords:** pressure ulcer, pressure injury, machine learning, MIMIC database, MIMIC-IV, open data, artificial neural network, random forest

## Abstract

Increasingly available open medical and health datasets encourage data-driven research with a promise of improving patient care through knowledge discovery and algorithm development. Among efficient approaches to such high-dimensional problems are a number of machine learning methods, which are applied in this paper to pressure ulcer prediction in modular critical care data. An inherent property of many health-related datasets is a high number of irregularly sampled time-variant and scarcely populated features, often exceeding the number of observations. Although machine learning methods are known to work well under such circumstances, many choices regarding model and data processing exist. In particular, this paper address both theoretical and practical aspects related to the application of six classification models to pressure ulcers, while utilizing one of the largest available Medical Information Mart for Intensive Care (MIMIC-IV) databases. Random forest, with an accuracy of 96%, is the best-performing approach among the considered machine learning algorithms.

## 1. Introduction

Pressure ulcers (PUs), also called pressure injuries (PIs), are classified into the category of non-healing or complicated healing wounds in most cases [1,2]. PUs burden not only the patients (necessity of wound care, pain, limited social interactions and a consequently worsening psychological status, etc.) but also represent a significant financial load on the health care services/systems (hospital, home care, caregivers, etc.). Non-healing wounds often reflect comorbidity or multimorbidity and represent the so-called silent epidemic affecting a large proportion of the world’s population [3].

The incidence of pressure injuries worldwide and the prevalence of pressure injuries in healthcare settings ranges from 0% to 72.5% [4,5,6,7]. It is estimated that around 10% of hospital patients and 5% of community care patients suffer from PUs and that 72% of all PUs occur in persons older than 65 years [8,9]. Differences in prevalence and incidence statistics are influenced by data collection and analysis methodology [10,11]. In the Czech Republic, there are two main sources for PUs monitoring. In the national adverse event reporting, the PUs are reported from all inpatient healthcare providers nationwide. The Adverse Event Reporting System (AERS) in the Czech Republic monitors the adverse events’ (AEs) occurrence in clinical practice and the subsequent data transmission to the central system [12]. The methodological documents are regularly updated, and the national AERS online portal was created [12] as a professional communication platform for healthcare providers (HCP). The data are reported by nurses or quality managers. AERS is a convenient tool for cultivating the quality of care at a national level, with data mandatorily provided by all inpatient facilities. The most commonly reported AEs for each reporting period were pressure ulcers—PUs (*n* = 48,704/2018; *n* = 48,779/2019; *n* = 47,755/2020). The data reported based on the ICD-10 codes are collected in the National Registry of Reimbursed Health Services (NRRHS). This registry is part of the National Health Information System (NHIS) and serves as a database where patients are reported to health care providers. The database contains data from health insurance companies, including complete data on reported diagnoses, procedures, and treatments. Patients with PUs are all diagnosed with L89* in the primary or secondary position on any medical document in a given year. Between 2010 and 2019, an average of 26,444 PUs per year were identified. In 2019, a diagnosis of L89 was reported in 30,590 patients, or 287 cases per 100,000 population. Most patients were reported to have category II (26.1%) or category III (23.9%) of all PUs [13]. Data analysis showed an increasing trend in PUs reporting, which may be related to improved ability to identify PUs and certainly to reimbursement of care for the higher PUs category.

Pressure ulcers are defined, according to the latest edition (2019) of the International Guideline for “Prevention and Treatment of Pressure Ulcers/Injuries”, as a localized damage to the skin and/or underlying tissue, as a result of pressure along or in combination with shear forces [2,14]. Prolonged pressure or shear results from contact between a bony prominence and the base or layer (bed, wheelchair, etc.). It predominantly concerns sacral, hip-trochanteric, and ischial areas. PUs can occur in various clinical forms, from non-blanchable skin erythema, superficial defects with affected subcutaneous tissue, and fistulas, to deep extensive defects with damaged muscles or bones. Clinical appearance of PUs is the basis for its classification—reversible PUs (category I and II), irreversible PUs (category III and IV), unstageable PUs, deep-tissue PUs, and specific PUs (medical device-related PUs and mucosal membrane PUs). [15] The pressure ulcer’s category determines its specific treatment—namely, conservative or surgical.

Prevention, early diagnosis, and adequate treatment play the most important role in skin and wound care in patients who are predisposed to PUs or who already suffer from them. Prevention goes hand-in-hand with an assessment of controlling risk factors of PUs; therefore, many assessment scale systems and tools were established to evaluate them. The Braden, Norton, or Waterloo scale systems are mostly used in clinical practice and are focused on moisture, incontinence, nutrition, mobility of the patient, etc. [16,17]. Superficial PUs (category I and II) are often omitted in lists of primary (main/principal) and secondary diagnoses during hospitalization. On the other hand, deep PUs (category III or IV) represent a serious complication that may increase the mortality of the patients, especially in intensive care units (ICUs) [18,19].

Therefore, establishing the predicting factors of PUs can help to eliminate the risk of the formation of hospital-acquired PUs (HAPUs). The formation and progression of PUs is affected by numerous factors; in other words, the causation is multifactorial. The determination of predictive factors, especially in case of hospital-acquired PUs and in critically ill patients in the ICU, can play an important role in their prevention. Predisposing factors for PUs formation are both intrinsic (comorbidities, poor nutritional status, limited mobility, etc.) [20,21] and extrinsic (excessive moisture, pressure from bed mattresses, shear forces from muscle spasms) [22]. In this paper, the following predictive factors of hospital-acquired PUs were included: basal constant demographic factors, such as age, gender and ethnicity.

Predictive factors related to gender are rather inconsistent. Kottner et al. presents that hospital-acquired PUs are a little more frequently found in men than in women. However, since this difference was slight, they concluded that gender should not be taken into consideration as an independent risk factor for PU development [23]. On the contrary, age is considered a basal risk factor of PUs formation [24]. It has been reported that up to 70% of PUs are found in patients aged 65 and older [25]. As far as ethnicity is concerned, Redelings et al. found that mortality related to PUs was higher among Black patients, as compared to Caucasians [26].

Other parameters studied over time were total intake, total output, arterial oxygen saturation, arterial systolic blood pressure, height, daily weight, glucose level, nutritional status parameters—albumin, total protein, and total bilirubin. Other predictive parameters were length of stay in bed and comorbidities concerning immobilization, such as spinal cord injuries and severe fractures. Other predictive factors were focused on local PUs assessment in correlation to the Braden score—sensory perception, moisture, activity, mobility, nutrition, and friction shear. At present, one of the main topics in the theoretical research on wound healing is the role of oxidative stress in various phases of the healing process [27]. In our further presented analyses, we did not find any parameters of oxidative stress identification. We can say, however, that it is still understandable, as although it is widely believed that the amount of oxygen/nitrogen radicals might be crucial for further direction of a healing process, there are several systematic studies presenting detailed insights into reactive oxygen species (ROS)/nitrogen species (RNS). However, their role in particular phases of wound healing is still limited. On the other hand, the parameters mentioned above are mostly clinically significant and well known in clinical practice.

This paper is unique in applying machine learning methods to pressure ulcer prediction in modular critical care data, utilizing the Medical Information Mart for Intensive Care (MIMIC-IV) database in particular. Rare instances of related work are discussed in the following sections of this manuscript and mainly concern qualitatively different databases, limited sample sizes, and different architectures of the machine learning algorithms.

The structure of the database, data selection criteria, and qualitative aspects of the healthcare data are described in Section 2. Machine learning algorithms and their application in medical research are detailed in Section 3. The results are discussed in terms of performance measures of selected classifiers, correlation and importance of input parameters, and confusion matrix terms.

The main concern of this paper is to address both theoretical and practical aspects related to the application of machine learning-based classification models to pressure ulcers, while utilizing one of the largest available healthcare datasets.

## 2. Materials and Methods

Pressure ulcers are statistically associated with different risk factors and preventive measures. The successful utilization of ML-based PU prediction models requires consistent reporting of clinical variable selections, data pre-processing, and model specifications. Ideally, ML models should be interpretable to allow clinicians to understand and improve model performance; however, according to a review from 2021 [28], only 2 out of 62 analyzed studies concerning the MIMIC dataset and the application of ML techniques in various ICU settings resorted to visualization-based interpretations. Traditional ML models can be more easily interpreted when compared to deep learning models with many levels of features and hidden layers. In [29], a multi-scale deep convolutional architecture has been proposed to tackle the problem of mortality prediction inside the ICU while offering interpretable predictions, i.e., predictions accompanied by explanations and/or justifications which make for a more transparent decision process. Here, not only dataset-level but also patient-level interpretability is provided, working with raw features instead of pre-processed ones; however, this study is focused on a more general topic of mortality prediction inside the ICU, when compared to the PU prediction.

As the predictor importance may differ significantly in time for any given patient, the sensitivity analysis of input features is nontrivial. Logistic regression can be used in combination with time-window averaging to identify important patient features; however, different resulting importance rankings represent an artifact of the selected time window.

In this study, time-varying patient features were averaged within a week-long time window (due to lack of data) before the first record of the PU for the PU group. For the non-PU group, this averaging was based on the first week after admission, in order to utilize this model in the future for objective assessments of special care requirements during admission.

Despite the increasingly available scientific computing clusters, the size of a typical medical database is prohibitive in terms of deep unsupervised learning, i.e., multivariate analysis of the entire database is not computationally feasible. This is due to not only memory requirements, but also data quality, as healthcare data are no longer small, structured, and collected exclusively in electronic health records.

Worldwide digital healthcare data is estimated to currently equal between 25 exa-bytes (25 × 10^18^ bytes) [30] and 35 zeta-bytes (35 × 10^21^ bytes) [30], with an annual increase of between 1.2 and 2.4 exabytes per year [30]. Such a huge amount of patient data is generated by a variety of lab systems and health information systems (e.g., EHRs, CPOE, PACS, CDSS).

According to Rehman et al. [31], the quality of healthcare data is a cause of concern for four reasons: incompleteness, inconsistency, inaccuracy, heterogeneity, and data fragmentation. A variety of techniques are required to analyze data quality, such as data standardization, verification, validation, monitoring, profiling, and matching. The problem of “dirty” data is mostly related to missing values, duplication, outliers, and stale records.

Due to the above-mentioned challenges, full-sensitivity and parametric studies are rarely conducted and input variables (patient features) as well as parameters (such as time windows) cannot be objectively (automatically) identified.

### Dataset

The data source for the presented study is the MIMIC-IV relational database, which represents the entire patient journey through a hospital, including performed procedures, medications given, laboratory values taken, and image analyses conducted [32]. This database is sourced from two in-hospital database systems, a custom hospital-wide electronic health record (HER) and an ICU-specific clinical information system. When creating the MIMIC-IV database, during the preparation process, data cleaning steps were not performed to ensure the data reflected a real-world clinical dataset. De-identifying results in date and time records random shifting into the future using an offset in days. Data for single patients are internally consistent; however, distinct patients are not temporally comparable [32].

A custom database for PU prediction has been extracted from MIMIC-IV, with 4652 patients with PU and a randomly sampled control group of the same size. Note that, due to the required normalization of the input variables, units are not relevant for the ML classification model.

Here, the time-invariant patient information includes age, gender, ethnicity, date of death, total intake (intravenous and fluid inputs), total output (patient outputs), and length of hospital stay.

The time-variant charted information includes arterial oxygen saturation, systolic arterial blood pressure, height, daily weight, and glucose (whole blood). The Braden scale [33] risk factors are also included sensory perception, moisture, activity, mobility, nutrition, and friction and shear. The nutritional assessment further includes albumin, total protein, and total bilirubin.

The patient information relating to fracture is a Boolean OR function that will result in TRUE if either one or more of the ICD-9 diagnosis codes related to fracture is present: fatigue fracture of vertebra; collapsed vertebra in diseases classified elsewhere; osteoporosis with pathological fracture; stress fracture, not elsewhere classified; pathological fracture, not elsewhere classified; fracture of bone in neoplastic disease; fracture of bone following insertion of orthopedic implant, joint prosthesis, or bone plate; fracture of skull and facial bones; fracture of neck; fracture of rib(s), sternum, and thoracic spine; fracture of lumbar spine and pelvis; fracture of shoulder and upper arm; fracture of forearm; fracture at wrist and hand level; fracture of femur; fracture of lower leg, including ankle; fracture of foot, except ankle; fractures involving multiple body regions; fracture of spine, level unspecified; fracture of upper limb, level unspecified; fracture of lower limb, level unspecified; and fracture of unspecified body region. 

**Figure 1:**
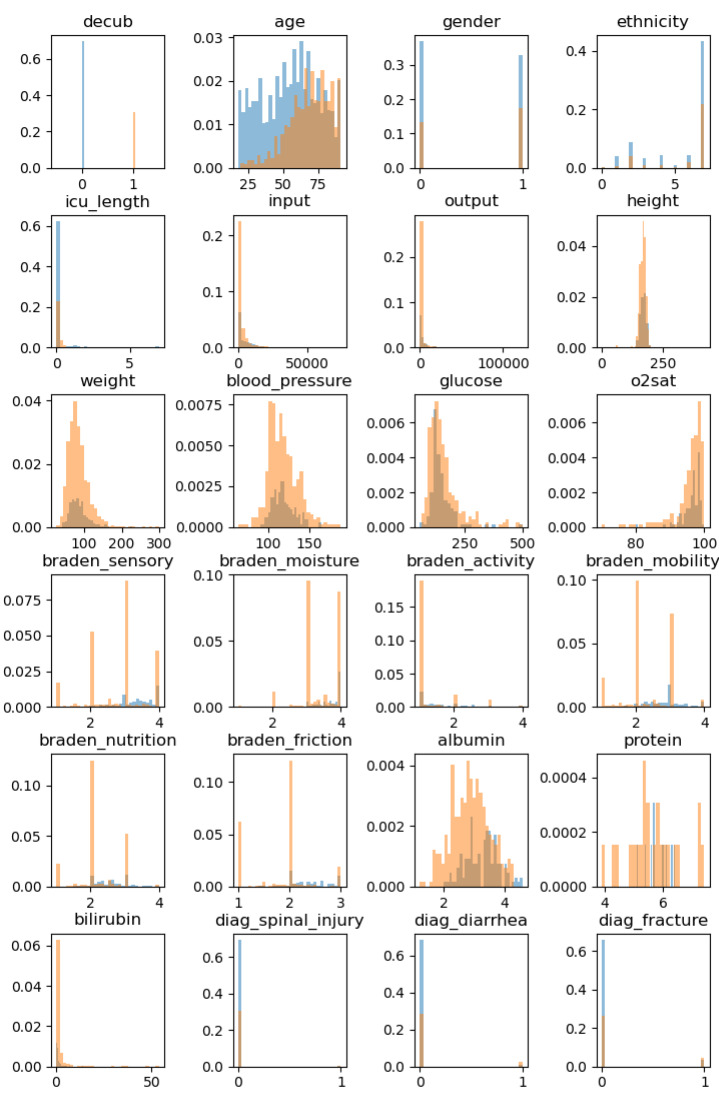
Histograms of non-debiased input parameters before normalization. The PU group is represented by blue color, while the orange color represents non-PU group.

**Figure 2:**
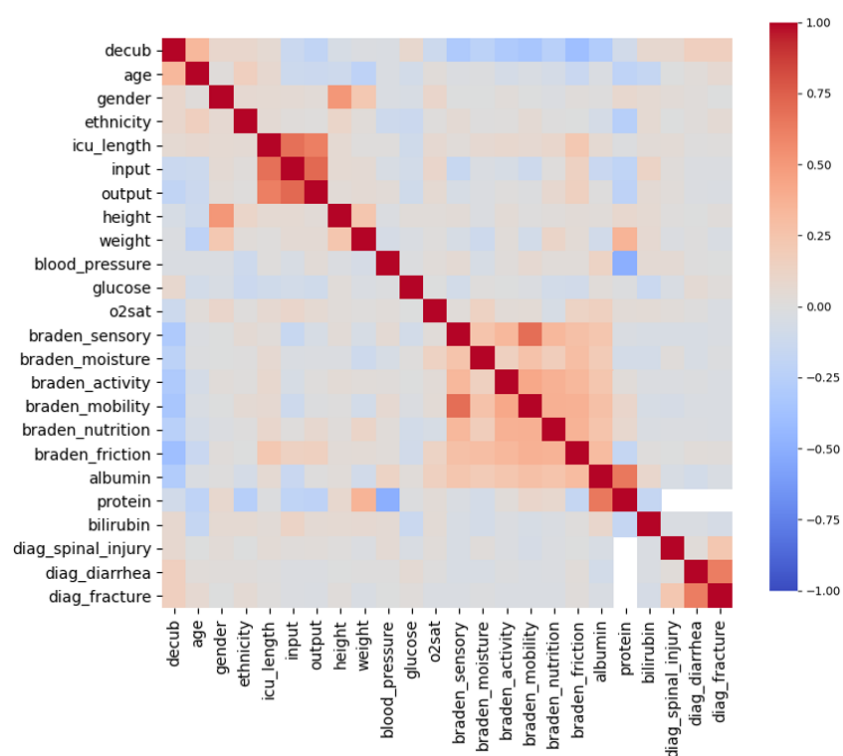
Correlation matrix of non-debiased input parameters.

Feature importance is computed as the mean and standard deviation of accumulation of the impurity decrease within each tree [34]. It is available both as an absolute value (FI) and a relative position (FI rank) in Table 1, together with a basic characterization of the input parameters, including the total count of PU patients and control group, their ratio, mean values, and variable type. The 4652 records of PU patients could not be used for the analysis due to the application of exclusion criteria. Patients had to be excluded if they died during hospital stay had an unrecorded PU date or had a majority of missing or null values in the selected input parameters. Debiasing [35] was used to tackle the sparsely populated data in included patients. As can be seen in Table 1, most patient features were not complete. Histograms of non-debiased input parameters before normalization are depicted in Figure 1. Correlation matrix (assuming linear relationship) for the input variables can be seen in Figure 2.

Error minimization is the usual goal of supervised machine learning classifiers while the choice of error evaluation metric is subjected to continuous debate in research and industry for several decades. A number of criteria need to be considered when choosing such a metric, e.g., interpretability, computational cost, differentiability, or popularity in a specific field.

## 3. Machine Learning Methods

It is well accepted that no classification method is universally better than any other [36]. Clearly, there are classes of target functions for which a method is best suited, and therefore, a cross-section of popular machine learning techniques has been chosen in order to predict the presence of pressure ulcers from a number of demographics and observed and measured patient features, with some characteristics unequally sampled in time (see Table 1). The medical data have been retrospectively collected within the MIMIC project [32].

Among the considered ML techniques are regression algorithms (logistic regression), instance-based algorithms (*k*-nearest neighbors and support vector machines), ensemble algorithms (random forest), artificial neural network algorithms (multi-layer perceptron), and Bayesian algorithms (naïve Bayes).

### 3.1. Regression Algorithms

Logistic regression (LR) is frequently used in medical research, as it estimates the relationship between one or more independent variables and a binary (dichotomous) outcome variable, such as “presence versus absence of pressure ulcer”, “dead versus alive”, or “positive versus negative for hypoxemia”. An example of multivariate logistic regression application to identify pressure ulcer risk factors can be found in [37].

The LR classification model assumes L2 regularization, also known as ridge regression. This technique is used to prevent overfitting by introducing a regularization term into the optimization problem. Tolerance is set to 10^−4^, the inverse of regularization strength (C) is set to 1.0, and the maximum number of iterations is limited to 100.

### 3.2. Instance-Based Algorithms

Space–time clusters of health events and their interactions are often investigated using the *k*-nearest neighbors (KNN) statistic, which is the number of case pairs that are *k*-nearest neighbors in both space and time, and is evaluated under the null hypothesis of independent space and time nearest neighbor relationships. Example applications can be found, e.g., in [38], where an adaptive-weighted *k*-nearest neighbors algorithm for the imputation of the first three months of screening visits has been developed.

The KNN model assumes a *k* parameter equal to 5 (based on heuristic technique), as larger values reduce the effect of noise on the classification, but make boundaries between classes less distinct. Additionally, the accuracy of KNN can be severely degraded if noisy or irrelevant features are present, or if the feature scales do not match their importance. Therefore, all input variables (patient features) were transformed to Gaussian distributions with zero mean value and unit standard deviation for all ML methods considered in this paper, assuming the central limit theorem.

According to [39], support vector machine (SVM)- and artificial neural network (ANN)-based classifiers have been the most useful artificial intelligence techniques to classify cancer. In particular, a study on liver biopsy images using a probabilistic neural network (PNN) has been presented, e.g., in [40]. An ANN classifier has also been used for breast cancer classification in the Wisconsin Breast Cancer Database (WBCD) [41], where a neural network with a feed-forward back-propagation algorithm was used to classify cancerous tumors from a symptom that causes the breast cancer disease. ANN classifiers are also used for successful lung cancer detection; in [42], a 16 descriptive attributes yield reported an accuracy of 97%. Based on various studies on cancer detection, SVM has the highest capability to classify datasets with a smaller number of input features, while ANN has better performance of accuracy in classifying datasets with a larger number of input features [39].

### 3.3. Artificial Neural Network Algorithms

The difference between ANN and SVM mainly concerns the classification of non-linear data, where SVM utilizes non-linear mapping to make the data linear separable, and therefore, the selection of the kernel function is the key. ANN, however, employs multi-layer connection and various activation functions in order to solve non-linear problems. Moreover, the more data is fed into the network, the better the generalization; thus, fewer errors can be expected from ANN. Conversely, SVM and random forest (RF) require significantly fewer input data.

The SVM model assumes a linear kernel with C equal to 2.0 and tolerance 10^−3^. The multi-layer perceptron (MLP) neural network model assumes two hidden layers (100 and 20), a rectified linear unit (ReLU) activation function (default activation function of many types of neural networks), and an Adam optimizer, which is invariant to diagonal rescales of the gradients and is appropriate for problems with noisy and sparse gradients [43]. The learning rate for MLP is set to 10^−3^ and the number of complete passes through the training dataset (epochs) is set to 300.

### 3.4. Bayesian Algorithms

A naïve Bayes (NB) classifier is used in [44] to detect cardiovascular disease and identify its risk level, consisting of a training set of tuples and their associated class labels. Here, the probability for a particular (cardiovascular) disease, given its symptoms, can be estimated using the Bayesian conditional probability model. In [45], a disease prediction system based on NB is presented, including typhoid, malaria, jaundice, tuberculosis, and gastroenteritis. NB is known for its limitation stemming from the assumption of independent predictors, which are almost absent in real-life scenarios; however, as a simple and fast method, NB is useful for real-time predictions, multi-class predictions, or recommendation systems in general.

### 3.5. Ensemble Algorithms

A random forest classifier has been successfully applied in healthcare monitoring systems in combination with the Internet of Things (IoT) in [46] to identify fraudulent behaviors in healthcare claims [47], or in evaluations of patient safety culture [48]. An RF model assumes 100 estimators and a maximal depth equal to 6, i.e., the number of trees in the forest and the maximal number of levels in each decision tree. According to [49], RF has the best accuracy in pressure ulcer prediction when compared to SVM, ANN, and decision tree (DT) models. This is in line with the conclusion of this paper, despite that the origin of the patients and the selected features are different.

## 4. Results and Discussion

**Figure 3:**
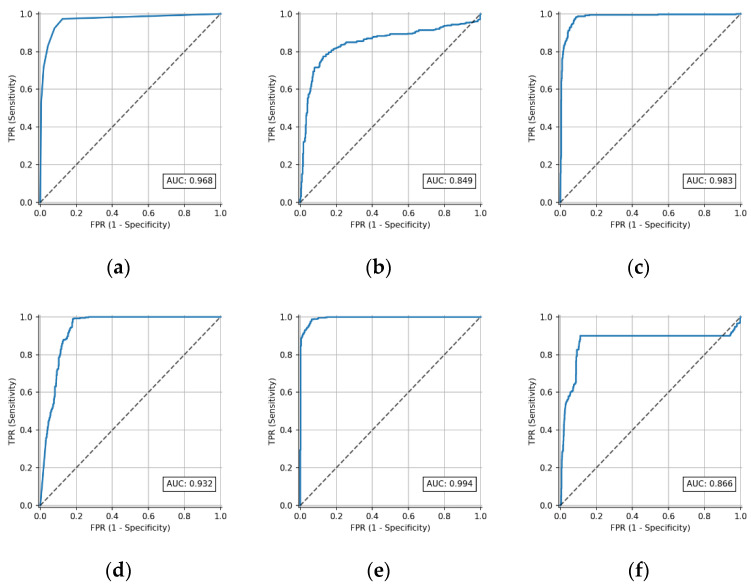
Performance of the 6 classification models considered at all classification thresholds (ROC curves): (**a**) *k*-nearest neighbors, (**b**) logistic regression, (**c**) multi-layer perceptron, (**d**) naïve Bayes, (**e**) random forest and (**f**) support vector machines.

Among the commonly used performance measures of classifiers based on machine learning methods are the receiver operating characteristic (ROC) curves and area under the ROC curve (AUC); see Figure 3. The raw data produced by a classification scheme during testing are counts of the correct and incorrect classifications from each class. This information is typically displayed in a *confusion matrix* (Table 2), which is a form of contingency table showing the differences between the true and predicted classes for a set of labelled examples [50].

While the ROC curve, which has been long used in conjunction with the Neyman–Pearson method [51] in signal detection theory, is a good visualization of a classifier’s performance; e.g., as a decision threshold or suitable operating point, often it is desirable to obtain a scalar measure, especially for cross-validated estimates of a classifier’s overall accuracy, i.e., the probability of a correct response. Such a single-figure estimate could be based on the area under the curve (AUC), or other popular metrics such as accuracy, precision, recall and F1-score; however, such measures are often insufficient, as they fail to characterize the complexity in model behavior, which has risen sharply over the last decade. For more thorough evaluation of classification models by probabilistic extension of the widely used threshold-based metrics, refer to [52].

Table 2 compares the above-mentioned metrics for the six considered ML methods and includes the average training times. The metrics are evaluated by standard binary classification with 0.5 threshold, i.e., accuracy is the fraction of correctly classified samples to total number of samples. Precision is the ratio of samples correctly classified to a particular class *c* to samples classified as class *c*, while recall is the fraction of samples in class *c* that are correctly retrieved. F1-score is an indicator quantifying the accuracy of a dichotomous model and it assumes both precision and recall of classification, i.e., it can be considered as a weighted average of model precision and recall. 

The selection of the RF model and its accuracy corresponds to a study from a Chinese hospital [49], where slightly fewer patients (85%) were included in the study, which also differed in a number of additional aspects. The RF model is also recommended in a similar study from the USA [53], where 39% of patients were included and the performance (AUC) reached 79%, when compared to results presented in this paper; however, stage I and stage II pressure ulcers were distinguished in the prediction, which surely resulted in the lower AUC.

A comprehensive review of the scientific literature concerning the use of ML algorithms for PU prevention has recently been published by [54], where the best-performing technique for the prediction of surgery-related pressure ulcers is ANN, with an accuracy of 81.5%.

This paper is unique in addressing both theoretical and practical aspects related to the application of ML models to pressure ulcers, while utilizing one of the largest available Medical Information Mart for Intensive Care (MIMIC) datasets. Given the size of the database, a big data approach is necessary and overfitting remains a challenge, given the high-dimensionality of the problem, as the number of available parameters, some of which are non-uniformly distributed (sampled) in time, is often equal to or greater than the number of patients, which can be included. This leads to the subjective choices regarding inclusion and exclusion criteria, which has to be realistically assessed given the available (and missing) data and the flexibility of the ML models.

Future work will include a distinction between more pressure ulcer groups and ML-based image processing and pattern recognition, towards automated and objective pressure ulcer classification.

In order to succeed, in general, the lag between data collection and processing has to be addressed, as well as the issues of ownership, governance, and standards. Moreover, health care data is rarely standardized, often fragmented, and is generated in legacy IT systems. This represents a major barrier in front of real-time big data analytics in performance-based healthcare systems.

## 5. Conclusions

The presented paper concerns the machine learning approach to pressure ulcer prediction based on a number of demographics and observed and measured patient features, retrospectively collected within the MIMIC project.

A cross-section of popular learning algorithms has been selected such that it represents various approaches to supervised ML, as up to the current date, there has been no classification method universally better than any other.

The best-performing approach among the considered ML techniques, which include regression algorithms, instance-based algorithms, ensemble algorithms, artificial neural network algorithms, and Bayesian algorithms, is random forest, yielding an accuracy of 96%.

The predictor importance differs significantly in time for any given patient and based on the sensitivity analysis of the input features of the best performing RF model. The most important patient features are ICU length of stay, total intake (intravenous and fluid inputs), and total output, i.e., time-invariant patient information that is independent from the time-window averaging scheme.

## Figures and Tables

**Table 1:** Characterization of input parameters and their importance for best performing RF model.

Parameter	Count dec	Count ndec	Ratio ndec/dec	Mean dec	Mean ndec	Data Type	FI	FI Rank
age	1979	4497	2.27	n/a	n/a	int64	9.41 × 10^−3^	12
gender	1979	4497	2.27	n/a	n/a	category	3.64 × 10^−4^	21
ethnicity	1979	4497	2.27	n/a	n/a	category	1.01 × 10^−3^	19
ICU length	1979	4497	2.27	0.37	0.25	float64	2.72 × 10^−1^	1
input	1979	793	0.40	2.69 × 10^3^	4615.48	float64	1.27 × 10^−1^	3
output	1952	784	0.40	1.38 × 10^3^	3543.23	float64	1.73 × 10^−1^	2
height	1356	442	0.33	168.33	170.02	float64	1.59 × 10^−2^	11
weight	1472	475	0.32	84.25	85.23	float64	3.09 × 10^−2^	9
blood pressure	413	185	0.45	118.23	119.24	float64	2.60 × 10^−3^	15
glucose	311	149	0.48	159.25	148.07	float64	2.84 × 10^−3^	13
o2sat	224	112	0.50	95.35	96.39	float64	1.27 × 10^−3^	17
Braden sensory	1519	456	0.30	2.73	3.31	float64	3.37 × 10^−2^	8
Braden moisture	1518	456	0.30	3.36	3.66	float64	4.33 × 10^−2^	7
Braden activity	1518	456	0.30	1.21	1.65	float64	1.17 × 10^−1^	4
Braden mobility	1517	456	0.30	2.28	2.85	float64	6.28 × 10^−2^	6
Braden nutrition	1517	456	0.30	2.16	2.51	float64	7.85 × 10^−2^	5
Braden friction	1513	456	0.30	1.80	2.36	float64	2.13 × 10^−2^	10
albumin	344	138	0.40	2.88	3.27	float64	2.60 × 10^−3^	16
protein	23	9	0.39	5.65	5.81	float64	2.88 × 10^−4^	22
bilirubin	491	188	0.38	1.74	1.06	float64	2.71 × 10^−3^	14
diag. spinal injury	1979	4497	2.27	n/a	n/a	bool	7.30 × 10^−5^	23
diag. diarrhea	1979	4497	2.27	n/a	n/a	bool	1.04 × 10^−3^	18
diag. fracture	1979	4497	2.27	n/a	n/a	bool	5.97 × 10^−4^	20

FI, feature importance; dec, patients with PU; ndec, patients without PU.

**Table 2:** Evaluation of machine learning algorithms: scalar performance measures and confusion matrix terms. Values are color-coded on a green (favorable values)-to-red (adverse values) scale.

	Accuracy	Precision	Recall	F1-Score	AUC	Time [s]				
Model		PPV	TPR				TPR	TNR	FPR	FNR
Random Forest	0.960	0.946	0.916	0.930	0.947	0.437	0.92	0.98	0.02	0.08
Multi-layer Perceptron	0.944	0.899	0.911	0.905	0.934	24,130	0.91	0.96	0.04	0.09
*k*-Nearest Neighbors	0.921	0.890	0.832	0.860	0.895	0.001	0.83	0.96	0.04	0.17
SVM (linear kernel)	0.873	0.785	0.779	0.782	0.845	7.825	0.78	0.91	0.09	0.22
Naïve Bayes	0.851	0.752	0.734	0.743	0.817	0.004	0.73	0.90	0.10	0.27
Logistic Regression	0.842	0.816	0.595	0.688	0.770	0.042	0.59	0.94	0.06	0.41

Based on 80:20 split and fixed seed. PPV, positive predictive value; TPR, true positive rate; TNR, true negative rate; FPR, false positive rate; FNR, false negative rate.

## Data Availability

This article is focused on anonymous data and information collection from EHR (Electronic Health Records) from database MIMIC IV (Medical Information Mart for Intensive Care). This database is open only for person, who has finished the Collaborative Institutional Training Initiative examination in Human Research-Data or Specimenens Only Research (Certification number 43354586 for corresponding author).
